# Determinants of gender disparities in scaling up the first 90 towards the UNAIDS 90–90–90 targets in South Africa: findings from the 2017 household-based national cross-sectional survey

**DOI:** 10.1186/s12981-021-00346-y

**Published:** 2021-04-28

**Authors:** S. Jooste, M. Mabaso, M. Taylor, A. North, Y. L. Shean, L. C. Simbayi

**Affiliations:** 1 Human and Social Capabilities Research Division, Human Sciences Research Council, 118 Buitengracht St, Cape Town City Centre, Cape Town, 8000 South Africa; 2 School of Nursing and Public Health, University of KwaZulu-Natal, Durban, South Africa; 3 Deputy CEO for Research, Human Sciences Research Council, Cape Town, South Africa; 4 Department of Psychiatry and Mental Health, University of Cape Town, Cape Town, South Africa

**Keywords:** HIV testing and awareness, 90–90–90 UNAIDS targets, Gender, South Africa

## Abstract

**Background:**

The first 90 of UNAIDS 90–90–90 targets to have 90% of the people living with HIV know their status is an important entry point to the HIV treatment cascade and care continuum, but evidence shows that there is a large gap between males and females in this regard. It is therefore important to understand barriers and facilitators of achieving the first 90 target. This study examined determinants of the first 90 target among females and males in order to inform strategies aimed at improving the HIV cascade in South Africa.

**Methods:**

The data used in the analysis were obtained from a 2017 household-based cross-sectional nationally representative survey conducted using a multi-stage stratified cluster random sampling design. A series of hierarchical multiple logistic regression models were fitted to identify the determinants of the first 90 target by gender.

**Results:**

Overall, 84.8% of HIV-positive individuals aged 15 years and older were aware of their HIV status. Females were significantly more aware of their HIV status compared to males (88.7% vs 78.2%, p < 0.001). Both females aged 25 to 49 years [aOR = 3.20 (95% CI 1.35–7.57), p = 0.008], and 50 years and older [aOR = 3.19 (95% CI 1.04–9.76), p = 0.042] and males aged 25 to 49 years [aOR = 3.00 (95% CI 1.13–7.97), p = 0.028], and 50 years and older [aOR = 7.25 (95% CI 2.07–25.36), p = 0.002] were significantly more likely to know their HIV status compared to those aged 15 to 19 years. Males with tertiary education level were significantly more likely to be aware of their HIV positive status [aOR = 75.24 (95% CI 9.07–624.26), p < 0.001] compared to those with no education or with primary level education. Females with secondary [aOR = 3.28 (95% CI 1.20–8.99), p = 0.021] and matric [aOR = 4.35 (95% CI 1.54–12.37), p = 0.006] educational levels were significantly more likely to be aware of their HIV positive status, compared to those with no education or with primary level education.

**Conclusion:**

Significant progress has been made with regards to reaching the UNAIDS first 90 target. In this context achieving the first 90 target is feasible but there is a need for additional interventions to reach the males especially youth including those with no education or low levels of education.

## Background

The Joint United Nations Programme on HIV/AIDS’ (UANIDS’) 90–90–90 strategy is to end the HIV epidemic by 2030 by achieving three targets, 90% of all people living with HIV know their status; 90% of all people diagnosed with HIV receive sustained antiretroviral therapy (ART); and 90% of all people on ART are virally suppressed [1]. The first 90 target is an important entry point to the HIV treatment cascade and care continuum [2]. This includes diagnosis and linkage to care, retention in care, adherence to ART, and viral suppression needed to remain healthy and live a long life with HIV [2, 3].

The South African government also adopted this strategy and has made tremendous progress towards the UNAIDS 90–90–90 targets, where knowledge of HIV status is the first step towards progress in the HIV cascade [2, 4, 5]. Although there has been a remarkable increase in HIV testing and awareness over the past decades more remains to be done to end the HIV epidemic by 2030. In sub-Saharan Africa (SSA), men account for 41% of people living with HIV and 53% of the AIDS-related deaths in 2016 were men [6]. Various socio-demographic, behavioural, and social characteristics have been associated with knowledge of HIV status. These include among others gender, age, marital status, educational level, employment status, socio-economic status, area of residence, stigma, and discrimination [5, 7, 8].

In Eastern and Southern African countries including South Africa, evidence points to large gaps between males and females in HIV testing and awareness including factors associated with the gender gap [5, 7, 9]. Understanding factors related to gender inequality in shaping the knowledge of HIV positive status is critical for designing interventions needed to bridge this gap, and for improving the HIV treatment and care cascade in South Africa. However, there is a paucity of nationally representative evidence. Therefore, more research is needed to understand the effects of various determinants related to gender inequality in influencing the testing and awareness of HIV status among people living with HIV. This paper examined the determinants of the first 90 target among females and males to inform strategies aimed at improving the HIV treatment and care cascade in South Africa.

## Methods

### Study data and sampling

The data used in the analysis were obtained from a nationally representative population-based household survey that was conducted in 2017 using a multi-stage stratified random cluster sampling design described in detail elsewhere [5]. A total of 1000 small area layers (SALs) were used as the primary sampling unit, drawn from the master sample through stratified, disproportionate sampling. The selection of SALs was stratified by province, locality type (urban areas, rural informal and formal areas), and race group. A total of 15 visiting points (VPs) were randomly selected from each of 1000 SALs, targeting 15,000 VPs. Of these, 12,435 (82.9%) VPs were approached. Among these VPs, 11,776 (94.7%) were valid VPs. A household response rate of 82.2% was achieved from the valid VPs (Simbayi et al. 2019). All consenting members of the selected household formed the ultimate sampling unit.

### Study procedure

The survey collected data using a household questionnaire and three age-appropriate questionnaires were administered to consenting individuals. For those younger than 18 years of age, consent was given by parents/guardians and assent by the participant. The interview instruments solicited information among others on socio-demographic characteristics, HIV-related knowledge, attitudes, and behaviours, including questions on HIV testing. The questionnaires were fieldworker administered and electronically captured using CSPro software on Mercer tablets. Fieldworkers also collected dried blood specimen samples from participants using a finger prick.

### HIV testing

Fieldworkers also collected dried blood specimen samples from participants using a finger prick. Samples were sent to a centralised laboratory for HIV antibodies testing using an algorithm with three different enzyme immunoassays (EIAs). All samples testing HIV positive during the first two EIAs (Roche Elecys HIV Ag/Ab assay, Roche Diagnostics, Mannheim, Germany and Genescreen Ultra HIV Ag/Ab assay, Bio-Rad Laboratories, California, USA) were subjected to a nucleic acid amplification test (COBAS AmpliPrep/Cobas Taqman HIV-1 Qualitative Test, v2.0, Roche Molecular Systems, New Jersey, USA) for the final interpretation of test results. Testing for exposure to antiretroviral drugs (ARVs) in HIV-positive specimens was performed using High-Performance Liquid Chromatography (HPLC) coupled with Tandem Mass Spectrometry.

### Ethical consideration

The survey protocol was approved by the Human Sciences Research Council (HSRC) Research Ethics Committee (REC: 4/18/11/15), and both the Division of Global HIV and TB (DGHT) and the Center for Global Health (CHG) of the Centers for Disease Control and Prevention (CDC). Ethical clearance was also obtained from the University of KwaZulu-Natal’s Biomedical Research Ethics Committee (BE 646/18). Verbal or written informed consent was sought before undertaking both the behavioural data and blood specimen collection.

## Measures

### Dependent variable

The primary outcome variable, the first 90 of the UNAIDS 90–90–90 targets [1] was defined as people who have been diagnosed HIV positive in the central laboratory and knew their status or were exposed to antiretrovirals, dichotomized as diagnosed and aware of HIV status = 1 and diagnosed and not aware of HIV status = 0.

### Independent variables

Explanatory variables were socio-demographic and HIV related behavioural characteristics. Socio-demographic characteristics included age group (15–19, 20–24, 25–49, 50 years and older), race groups (African and other race groups), marital status (married, never married), level educational qualification (no education/primary, secondary, matric, tertiary), employment (yes, no), and locality type (urban areas, rural informal/tribal areas, rural formal/farms). HIV related behaviour characteristics included condom use last sex act (yes, no), correct HIV knowledge and myth rejection (yes, no), and self-perceived risk of HIV infection (yes, no).

### Statistical analysis

Descriptive statistics were used to summarize the sample characteristics. Proportion tests *“prtest”* command were used to test for differences between the explanatory variables and the first 90 target by gender. A series of hierarchical multiple logistic regression models structured by sex (males and females) were fitted, and the estimates of the contributions of each independent variable were computed against the dependent variable in successive models. The best-fitting models with variables that reliably predict the first 90 target were determined by assessing changes in R-squared (R2) values and best predictors by adjusted odds ratios (aOR) with 95% confidence intervals (CIs) and p ≤ 0.05. The ‘svy’ command was used to introduce weights that take into account the complex design of the survey. All data analyses were conducted using STATA version 15.0 (STATACORP, College Station, TX) software.

## Results

### Sample characteristics

**Fig. 1:**
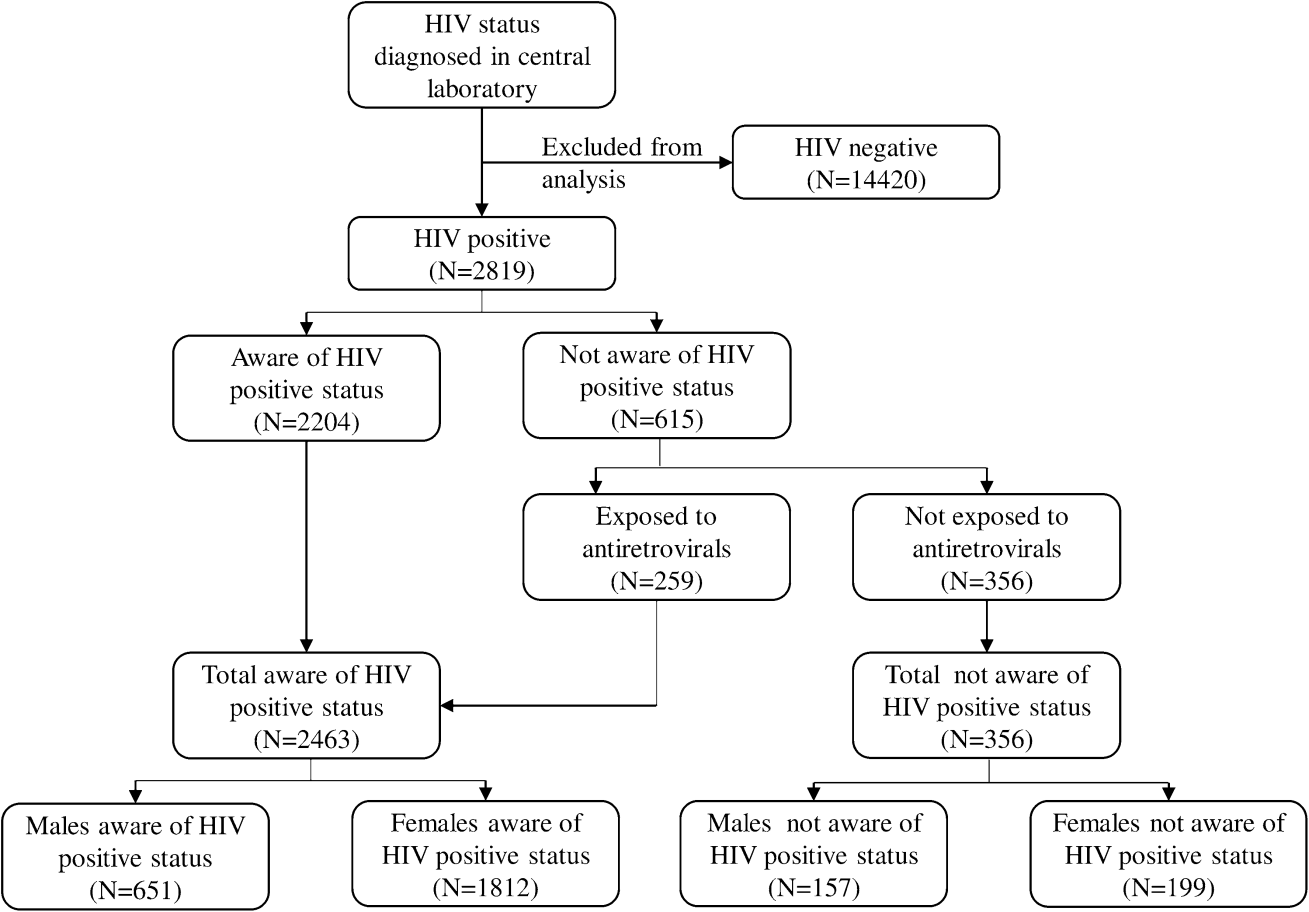
Description of first 90 (people living with HIV who know their HIV) by sex

Figure 1 shows the sub-sample of those who tested for HIV and the breakdown of the first 90 the primary outcome of interest in the study.

Table 1 shows the summary statistics of the study sample and the first 90 target. Of 2819 individuals (males, n = 808 and females, n = 2011) that tested HIV positive in the survey. The majority were aged 25 to 49 years of age (74.8%), were Black African (96.1%), were not married (78.4%), were unemployed (66.2%), and resided in urban areas (62.2%). Less than half (45.2%), had completed secondary level education, more than half (52.3%) used a condom at last sex, while the majority did not have accurate knowledge about preventing HIV transmission and rejection of misconceptions (64.8%) and had a low self-perceived risk of becoming HIV infected (74.3%). The characteristics of males and females that tested HIV positive were similar to the overall sample except that more males were married (28.3% vs 17.8%) and employed (47.1% vs 26.2%) compared to females.

**Table 1:** Characteristics of the study sample aged 15 years and older who tested HIV positive and were aware of their status by gender, South Africa 2017

Variables	Total		Males		Females	
	n	%	n	%	n	%
Age categories						
15–19	110	3.2	36	3.9	74	2.8
20–24	226	6.9	51	4.4	175	8.3
25–49	1959	74.8	558	75.5	1401	74.5
50+	524	15.0	163	16.1	361	14.4
Race						
Black African	2573	96.1	730	94.9	1843	96.8
Other	246	3.9	78	5.1	168	3.2
Currently married						
Married	579	21.6	228	28.3	351	17.8
Not married	2065	78.4	525	71.8	1540	82.2
Education level completed						
No education/primary	592	20.4	193	21.2	399	19.9
Secondary level education	1075	45.2	312	46.7	763	44.3
Matric	590	28.9	144	25.7	446	30.7
Tertiary level education	118	5.6	35	6.4	83	5.1
Employment status						
Unemployed	1749	66.2	388	52.9	1361	73.9
Employed	865	33.8	358	47.1	507	26.2
Locality type						
Urban areas	1505	62.2	440	65.9	1065	60.1
Rural informal areas	899	30.7	198	23.2	701	35.1
Rural farms	415	7.1	170	11.0	245	4.9
Condom use last sex act						
No	778	47.8	225	46.1	553	48.8
Yes	765	52.3	249	53.9	516	51.2
Correct knowledge and myth rejection						
No	1701	64.8	477	65.1	1224	64.7
Yes	941	35.2	274	34.9	667	35.3
Self-perceived risk of HIV						
Low	1090	74.3	374	79.3	716	70.8
High	366	25.8	99	20.7	267	29.2

Table 2 shows that a large majority (84.8%) of the HIV positive sample were aware of their HIV status. Higher awareness of HIV status was found among those aged 25 to 49 years, Black African and people living in urban areas, those who were married, had matric or higher education, were unemployed and used a condom at last sex achieved the first UNAIDS 90 target. Females had a significantly higher awareness of HIV status compared to males (88.7% vs 78.2%, p < 0.001). Testing and awareness of HIV status among females relative to males were significantly higher by race, marital status, employment status, locality type, condom use at last sex, HIV knowledge, and risk perception. Females in the 15 to 19 year and 25 to 49 year age groups had significantly higher awareness than males in the same age groups (p < 0.001). Females with secondary and matric level education had a higher awareness of their HIV status than their male counterparts with the same level of education (p < 0.001).

**Table 2:** Sample characteristics of individuals diagnosed HIV positive and aware (UNAIDS’ first 90 target) among youth and adults 15 years and older by sex, South Africa 2017

Variables	Total sample diagnosed and aware			Males diagnosed and aware			Females diagnosed and aware			
	N	%	95% CI	n	%	95% CI	n	%	95% CI	p-value
Total	2819	84.8	81.6–87.6	808	78.2	72.4–83.1	2011	88.7	85.7–91.1	<0.001
Age categories										
15–19	110	75.2	63.6–84.0	36	62.8	44.0–78.5	74	85.1	73.7–92.1	0.008
20–24	226	73.4	59.4–83.9	51	73.2	56.0–85.4	175	73.5	55.6–86.0	0.966
25–49	1959	85.8	82.2–88.8	558	77.6	70.7–83.3	1401	90.6	87.0–93.4	<0.001
50+	524	87.3	81.4–91.5	163	86.2	76.0–92.4	361	88.0	82.0–92.2	0.565
Race										
Black African	2573	85.3	82.1–88.0	730	78.8	72.8–83.8	1843	89	85.9–91.5	<0.001
Other	246	73.1	54.1–86.2	78	66.6	43.4–83.9	168	79	60.2–90.4	0.036
Marital status										
Married	579	91.8	87.8–94.6	228	87.4	79.6–92.4	351	95.8	92.6–97.7	<0.001
Not married	2065	88.9	86.4–91.1	525	83.0	77.8–87.2	1540	91.9	88.9–94.2	<0.001
Education level										
No education	592	84.3	76.5–89.8	193	82.4	73.5–88.8	399	85.4	72.9–92.7	0.345
Secondary level	1075	89.9	86.9–92.2	312	82.6	75.9–87.8	763	94.3	91.8–96.0	<0.001
Matric	590	92.6	89.7–94.8	144	86.0	77.9–91.5	446	95.9	93.6–97.3	<0.001
Tertiary level	118	97.4	91.9–99.2	35	99.7	97.7–100.0	83	95.7	86.4–98.7	0.253
Employment status										
Unemployed	1749	90.0	87.3–92.2	388	85.7	80.7–89.5	1361	91.8	88.4–94.3	<0.001
Employed	865	88.9	85.6–91.6	358	83.3	77.1–88.1	507	94.7	92.0–96.6	<0.001
Locality type										
Urban areas	1505	87.7	84.5–90.2	440	82.8	77.1–87.3	1065	90.7	87.5–93.2	<0.001
Rural informal areas	899	85.8	80.3–89.9	198	79.7	68.5–87.6	701	88.1	81.7–92.5	0.002
Rural formal areas	415	56.2	37.7–73.1	170	47.3	27.8–67.7	245	67.7	51.3–80.6	<0.001
Condom use last sex act										
No	778	88.9	85.6–91.5	225	79.5	72.0–85.5	553	94.6	91.9–96.5	<0.001
Yes	765	94.3	91.4–96.2	249	89.8	83.3–94.0	516	97.3	95.1–98.6	<0.001
Correct knowledge and myth rejection										
No	1701	89.4	86.3–91.8	477	85.5	80.5–89.5	1224	91.6	87.7–94.3	<0.001
Yes	941	89.9	86.9–92.3	274	81.8	74.4–87.4	667	94.5	92.2–96.2	<0.001
Self-perceived risk of HIV infection										
Low	1090	81.5	77.1–85.1	374	75.8	68.7–81.7	716	85.9	79.7–90.4	<0.001
High	366	85.0	79.8–89.0	99	75.6	62.7–85.1	267	89.6	84.3–93.2	<0.001

### Hierarchical regression models

**Fig. 2:**
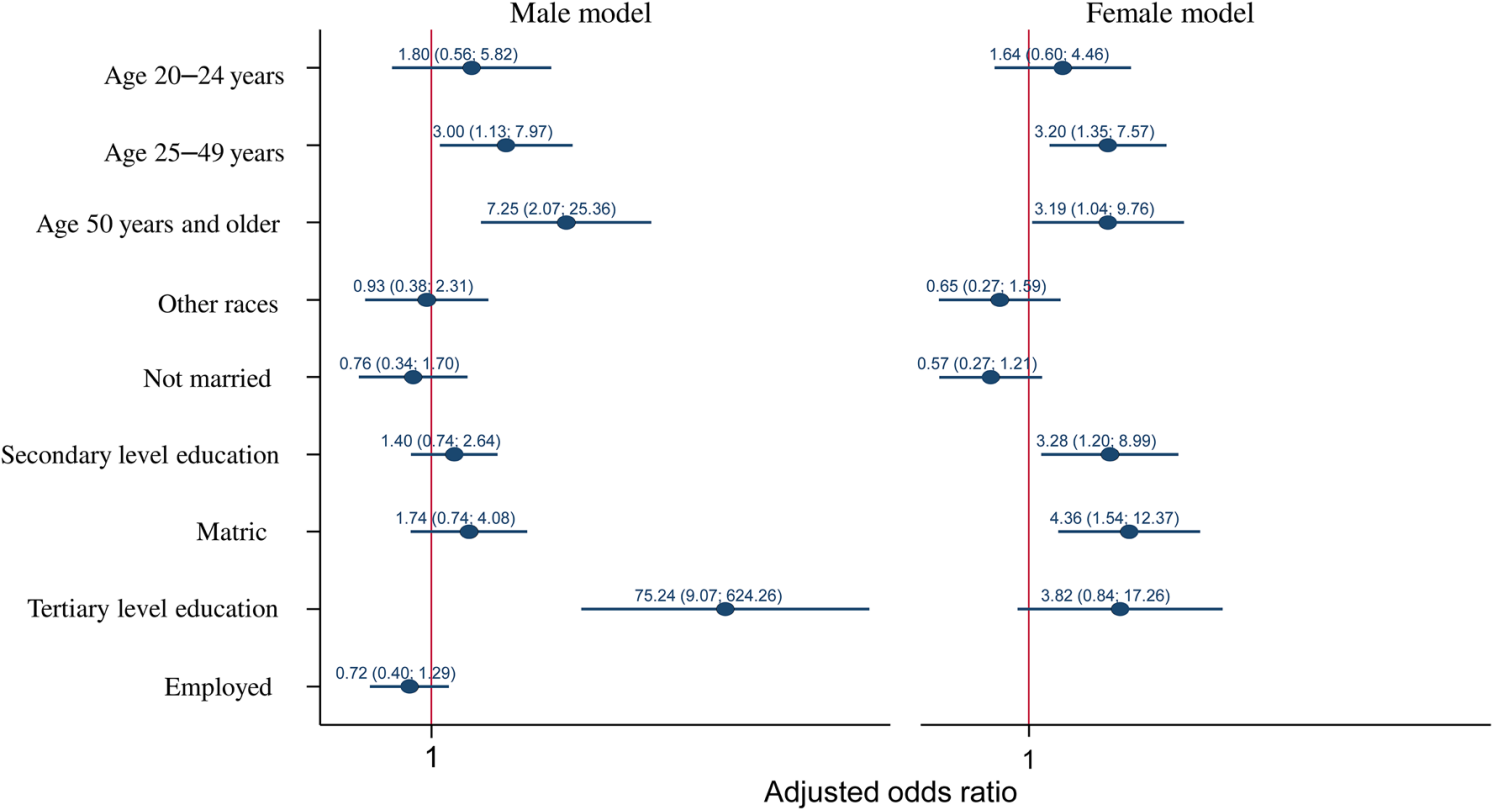
Hierarchical logistic regression models of factors associated with UNAIDS’ first 90 target, South Africa 2017

Figure 2 shows the results of the multiple hierarchical regression models of factors associated with testing positive for HIV and being aware of their status (UNAIDS’ first 90 target) among males and females aged 15 years and older. Males 25–49 years [aOR = 3.00 (95% CI 1.13–7.97), p = 0.028], those 50 years and older [aOR = 7.25 (95% CI 2.07–25.36), p = 0.002] were significantly more likely to be aware of their HIV positive status compared to those 15–19 years. In addition, males with tertiary educational qualifications were significantly more likely to be aware of their HIV positive status [aOR = 75.24 (95% CI 9.07–624.26), p < 0.001] compared to those with no education or with primary level education.

Similarly, females 25–49 years [aO = 3.20 (95% CI 1.35–7.57), p = 0.008] and those 50 years and older [aOR = 3.19 (95% CI 1.04–9.76), p = 0,042] were significantly more likely to be aware of their HIV positive status compared to those 15–19 years. Furthermore, females with secondary [aOR = 3.28 (95% CI 1.20–8.99), p = 0.021] and matric [aOR = 4.35 (95% CI 1.54–12.37), p = 0.006] educational qualifications were significantly more likely to be aware of their HIV positive status compared to those with no education or with primary level education

## Discussion

In this representative national survey, more than three quarters (84.8%) of the HIV-positive youth and adult population were already diagnosed at the time of the survey and were aware of their status. These results were higher than data from countries in other population surveys in the Eastern and Southern African sub-regions [10]. Overall, awareness of HIV positive status was higher among those aged 25 to 49 years, Black African and people living in urban areas, those who were married, had matric or higher education, were unemployed and used a condom at last sex, who achieved the first UNAIDS’ 90 target.

This study also confirmed that the proportion of youth and adults diagnosed and aware of their HIV positive status was significantly higher among females compared to males for all the above socio-demographic and socio-behavioural factors. Evidence shows that Black African females are more likely to have an HIV test in their lifetime than their male counterparts, due to their exposure to sexual reproductive health services [5, 8, 11, 12]. This suggests that greater exposure to HIV testing is associated with higher awareness of HIV status [8]. This implies that encouraging routine HIV testing and awareness may be especially challenging for males who are less likely than females to engage in preventive health care overall [5, 11, 12].

These findings are consistent with other studies conducted across sub-Saharan Africa (SSA). The 2015–2016 Malawi Population-based HIV Impact Assessment [6] found that men were 12% less like to know their HIV status than women, and a third lower in Niger and Paraguay among men living with HIV than women living with HIV. These findings point to harmful hegemonic gender norms and stereotypes that emphasise men’s invulnerability to illness, their masculinity and male dominance contribute to poor health outcomes and discourage them to know their status [13]. Gender, defined as the “socially constructed roles, behaviour, activities and attributes that a particular society considers appropriate for men and women” [14] has been identified as a social structure which is deeply embedded at all levels of society [15], and plays a central role in the HIV/AIDS epidemic [16]. Men were found to be resistant to HIV testing and prevention efforts [17] and that an HIV test was also seen as the impetus for change in behaviour and therefore undesirable among men [18].

The findings from logistic regression models indicated that a rather similar set of demographic factors were common among both males and females who tested HIV positive and were aware of their status in the current study. These findings suggest that age and education may influence HIV testing and awareness. Others found no significant association between gender and HIV testing and awareness but rather with age [19–21]. The youth were less likely to report having been tested and being aware of their status compared to the older age groups [19, 20]. Young people (15 to 25 years of age) were also more likely to engage in risky sexual behaviours regardless of gender, and so represent the target population for most interventions and campaigns to improve HIV testing and awareness [20].

The current focus of the South African government and international agencies is on reducing the burden of HIV amongst adolescent females living with HIV. This study revealed that the group not currently linked to HIV treatment are those who are not aware of their status, which is concerning given that the essential step in the HIV care pathway for HIV‐positive individuals starts with HIV testing [22]. This was worse among males compared to females with about 60% HIV positive adolescent males who were not aware of their status at a population level. In other words, more than a third of people living with HIV in this group could transmit the virus unless they are linked to care and provided with prevention tools and empowered to use them. The identified gap towards achieving the first 90 among adolescent males could not be ascertained using routine programme data. Therefore, this study provides vital strategic information on one of the key pillars of the 90–90–90 UNAIDS targets in the country.

Reaching the first UNAIDS target will require concerted efforts to increase the uptake of HIV testing by addressing accessibility of services through targeted self-testing, mobile and community-based testing [23–26]. Evidence suggests that the public nature of HIV testing campaigns influence men’s uptake of testing with more preference for home-based testing [27, 28]. This reduces stigma and vulnerability attributable to attitudes or assumptions regarding individuals who request HIV testing in public venues or health centres [27]. Such engagement can facilitate social and interpersonal support by trusted friends, partners, and family members. It is therefore important to determine preferences of men for HIV testing in order to inform programs regarding where to focus male HIV testing resources and achieve greatest impact [27]. The provision of adolescent-friendly services is another strategy for encouraging HIV testing uptake among young men and a key component of national HIV responses.

In agreement with the current findings, the evidence shows that in impoverished communities both youth and adult males and females with greater educational attainment (high school diploma or greater) were more likely to report having been tested for HIV and were aware of their status than those who had no education or had not graduated from high school [5, 11, 12]. This suggests that educational level may not only influence one’s likelihood to seek HIV testing but may also play a pivotal role in increasing awareness of ones’s HIV status. This finding underscores the importance of formal education which is often viewed as an essential factor in the social determinants of health, especially in disadvantaged communities. Improving the level of education will not only inform HIV testing and awareness in impoverished communities but can also positively influence linkage to treatment and care in these communities.

While addressing the educational needs of impoverished communities is a potential strategy to increase HIV testing, there is also a need for providing initiatives to overcome barriers to testing among those with no formal education. The educated might have higher levels of exposure to HIV-related information, better knowledge of the advantages of HIV testing as well as ability to make good decision to go for HIV testing than their uneducated counterparts. These observations highlight the importance of providing health education including HIV-related information to those with low level or no education. An important dimension of bridging this gap is equity, which can be achieved through community outreach to facilitate HIV testing uptake through home, mobile and community-based strategies. Promoting HIV testing through community meetings among those with no formal education could be a cornerstone towards closing the testing gap and achieving the first '90' target.

This study has some limitations that should be noted. Explanatory data used in the analysis was self-reported and could be subject to recall and social desirability bias. The sample of HIV-diagnosed individuals who were aware of their status was smaller, and consequently part of the analysis lacked precision which is reflected in the large confidence intervals which are reflected in some of the results. This study was cross-sectional and was only limited to ascertaining the association between the primary outcome and potential explanatory variables and could not infer causality. As with any observational investigation, the study is limited in the number of variables and there may be other, unmeasured factors that contribute to the observed associations. Nevertheless, this study was based on a nationally representative population sample and the findings can be generalised to individuals diagnosed with HIV and who are aware of their status.

## Conclusion

This study suggests an association between HIV testing and awareness with age and education level among both males and females as an important combination of factors that determine population groups and barriers that need to be targeted to make progress to achieving the first 90 of the UNAIDS targets towards ending HIV in 2030. The findings study revealed that the group not currently linked to HIV treatment are those who are not aware of their status, and this gap was huge among male adolescents compared to their counterparts as well as among those with little or no education. Substantial opportunities exist to optimize the uptake of HIV testing though innovative application of existing intervention strategies. A greater emphasis should be placed on promoting and increasing demand for the different HIV testing models as well as improving both formal and HIV related education as a means of reducing HIV testing and awareness disparities in the country towards improving the HIV treatment and care cascade in South Africa.

## Data Availability

The datasets analysed during the current study are available in the [SABSSM] repository, Human Sciences Research Council. *South African National HIV Prevalence, HIV Incidence, Behaviour and Communication Survey (SABSSM) 2017: Combined - All provinces*. [Data set]. SABSSM 2017 Combined. Version 1.0. Pretoria South Africa: Human Sciences Research Council [producer] 2017, Human Sciences Research Council [distributor] 2020. http://dx.doi.org/doi:10.14749/1585345902
